# Epstein-Barr Virus (EBV) Hepatitis Treated With N-Acetylcysteine: Placebo Versus True Effect

**DOI:** 10.7759/cureus.104922

**Published:** 2026-03-09

**Authors:** Ariel Ahl, Sandra Abadir, Garo Kalfayan, Kumar Desai

**Affiliations:** 1 Internal Medicine, Los Robles Regional Medical Center, Thousand Oaks, USA; 2 Gastroenterology, Los Robles Regional Medical Center, Thousand Oaks, USA

**Keywords:** acute viral hepatitis, ebv-associated hepatitis, epstein-barr virus, n-acetylcysteine (nac) therapy, n-acetyl cystine

## Abstract

Epstein-Barr virus (EBV) is a herpesvirus that is known to cause multiple conditions, ranging from malignancies like Hodgkin and non-Hodgkin lymphomas to infectious and autoimmune conditions like infectious mononucleosis and systemic lupus erythematosus. Infectious mononucleosis causes systemic symptoms including cervical lymphadenopathy, splenomegaly, malaise, myalgias, and fever. While the spleen and liver are involved in some cases, most cases result in mild elevation of transaminases, not exceeding two to three times the upper normal limit. The transaminitis is usually subclinical with a self-limiting course. On rare occasions, liver involvement is associated with significant transaminitis reaching up to 10 times the upper normal limit. These patients usually also present with jaundice. Such severe or fatal hepatitis is most commonly seen in immunocompromised individuals. Here, we present a 31-year-old man who came to us with progressive jaundice, upper quadrant abdominal pain, and greasy stools after what he thought was just the flu. The patient was found to have EBV-induced hepatitis, requiring N-acetylcysteine administration.

## Introduction

Epstein-Barr virus (EBV) is a herpesvirus that was discovered in 1964. The virus was initially associated with Burkitt lymphoma, but since then it has also been linked to Hodgkin and non-Hodgkin lymphomas, nasopharyngeal carcinoma, gastric cancers, multiple sclerosis, systemic lupus erythematosus, and infectious mononucleosis [1]. The virus is highly prevalent, with up to 90% of the population worldwide infected. While the main route of transmission is oral, several reports have documented transmission through blood transfusions and organ transplants [2].

Infectious mononucleosis (IM) is characterized by severe pharyngitis, adenopathy, malaise, myalgia, and fever and is observed mainly in young adults, lasting several weeks. Other symptoms like hepatomegaly, splenomegaly, abdominal pain, and a rash, especially after the administration of penicillin derivatives, have also been reported in less than 20% of the patients with IM. The hallmark of the condition is the presence of atypical or Downey cells seen on peripheral blood smears. While the condition is self-limiting, long-term effects such as the development of autoimmune conditions or malignancies are complications that need to be followed in these patients [3]. One of the most common and feared gastrointestinal complications seen with IM is splenic rupture; however, in some rare instances, moderate cholestatic hepatitis can occur, leading to a moderate elevation in transaminitis and jaundice [4]. N-acetylcysteine (NAC) is an antioxidant used in a variety of conditions, such as acetaminophen and heavy metal toxicity, bone regeneration, and even as a chemotherapeutic agent. Its antioxidant effect can also reduce the degree of inflammation caused by viruses and has been reported in some literature to facilitate the resolution of post-viral hepatitis [5]. Based on several recent reports, NAC has shown promise in reducing inflammation associated with viral hepatitis. In particular, several case reports have demonstrated its potential role in progressive EBV hepatitis and acute liver failure. Understanding the spectrum of complications in IM is crucial for timely management and follow-up.

## Case presentation

Our patient is a 31-year-old man with no medical history who presented to the emergency department (ED) due to progressive jaundice. Two weeks prior to admission, he was experiencing flu-like symptoms, including malaise, fatigue, joint pain, headaches, nausea, and vomiting. He additionally reported chills, painful swallowing, and enlarged cervical lymph nodes. His symptoms self-resolved three days prior; however, he subsequently developed left upper quadrant pain, greasy stools, and jaundice, which have since worsened.

The patient denied any history of recent travel. He stated that he went hiking two weeks prior but was unsure if he had any bug bites. He does not take any medications regularly. His only surgical history was an appendectomy as a child. He denied any history of IV drug use. The patient had an extensive alcohol use history and reported binge drinking daily in the past. We noted that over the past three months, he had regularly been drinking five to six beers in one sitting every weekend. He reported that his last drink was 72 hours before the onset of symptoms. The patient has been monogamous with his current partner; however, prior to this relationship, he was sexually active with multiple female partners, with consistent use of condoms. Of note, the patient works as an emergency medical technician (EMT) at a local hospital and stated that he is frequently in contact with needles; however, he was unsure of any needlestick accidents.

On presentation, the patient had a temperature of 99.1°F, heart rate of 83 beats/minute, respiratory rate 18 breaths/minute, oxygen saturation of 99% on room air, and blood pressure of 143/79 mm/hg. Physical examination was remarkable for bilateral scleral icterus and jaundice below the tongue. No cervical adenopathy was appreciated. Abdominal examination was without any tenderness, guarding, or rigidity.

Initial laboratory values were remarkable for elevated WBC, low sodium, elevated total bilirubin and direct bilirubin, elevated liver function tests, elevated lactate dehydrogenase, and a normal acetaminophen level (Table 1). Platelet count, prothrombin time (PT), and international normalized ratio (INR) were within normal range.

**Table 1:** Pertinent laboratory values on presentation, including blood counts, chemistry panel, and liver synthetic function

Laboratory Marker	Laboratory Value	Normal Range
WBC	16.6 x 10^3^/µL	4.0–11.0 x 10^3^/µL
Sodium	132 mmol/L	136–145 mmol/L
Aspartate transferase (AST)	261 IU/L	10–37 IU/L
Alanine transaminase (ALT)	383 IU/L	16–61 IU/L
Alkaline phosphatase (ALP)	345 IU/L	45–117 IU/L
Total bilirubin	6.1 mg/dL	0.2–1.0 mg/dL
Direct bilirubin	4.79 mg/dL	<0.2 mg/dL
Lactate dehydrogenase	685 IU/L	120–250 IU/L
Acetaminophen	<2 mcg/dL	N/A
Platelet	211 x 10^3^/µL	150–400 x 10^3^/µL
Prothrombin time	11.4 seconds	9.8–11.6 seconds
I*nternational normalized ratio (*INR)	1.08	N/A

**Figure 1:**
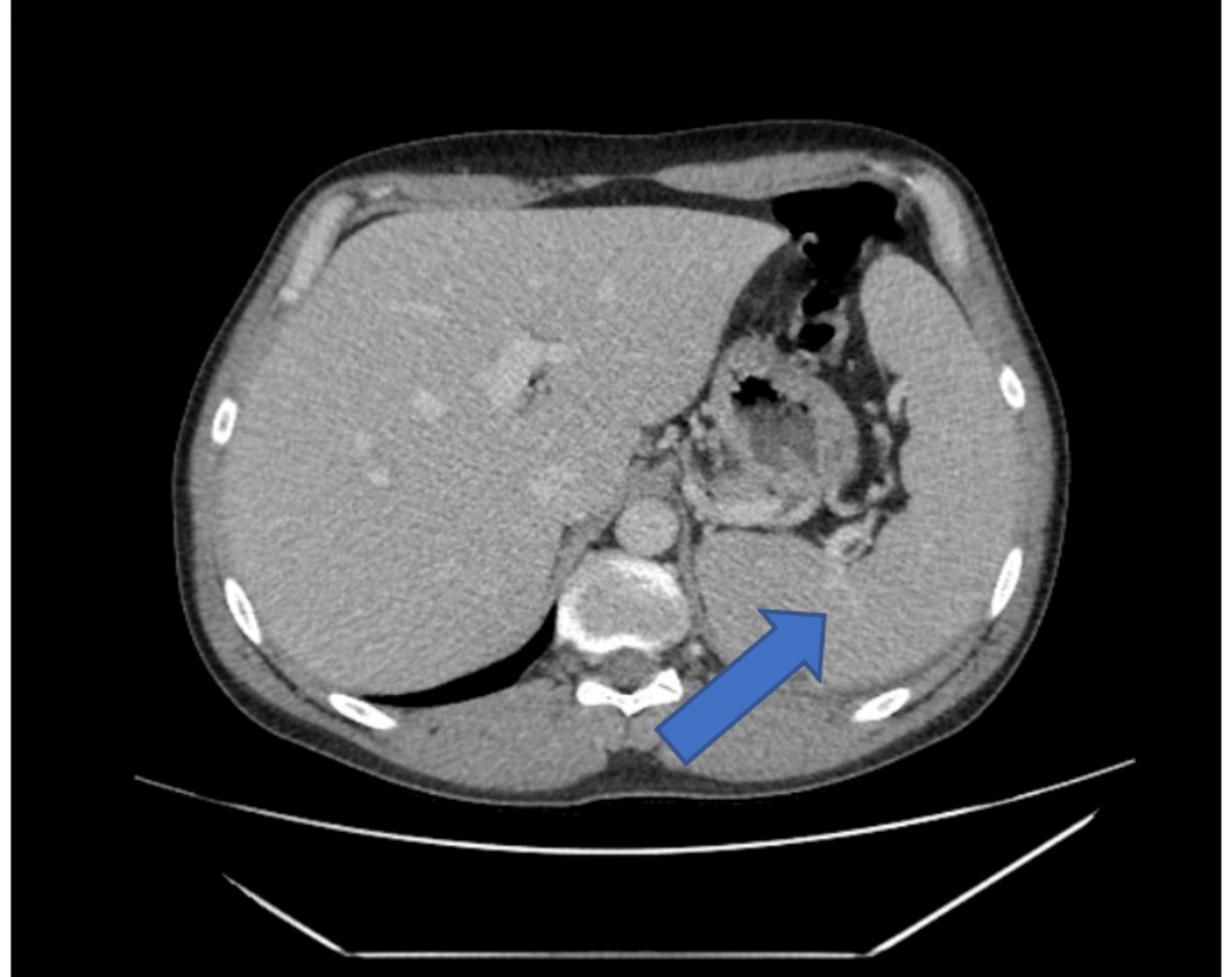
Computed tomography of the abdomen and pelvis demonstrating splenomegaly (blue arrow)

The acute viral hepatitis panel was negative. A peripheral blood smear showed lymphocytosis with normochromic and normocytic anemia but without increased myeloblasts. A rapid HIV test was negative. Abdominal ultrasound was significant for a nodular density near the pancreatic head (Figure 1).

**Figure 2:**
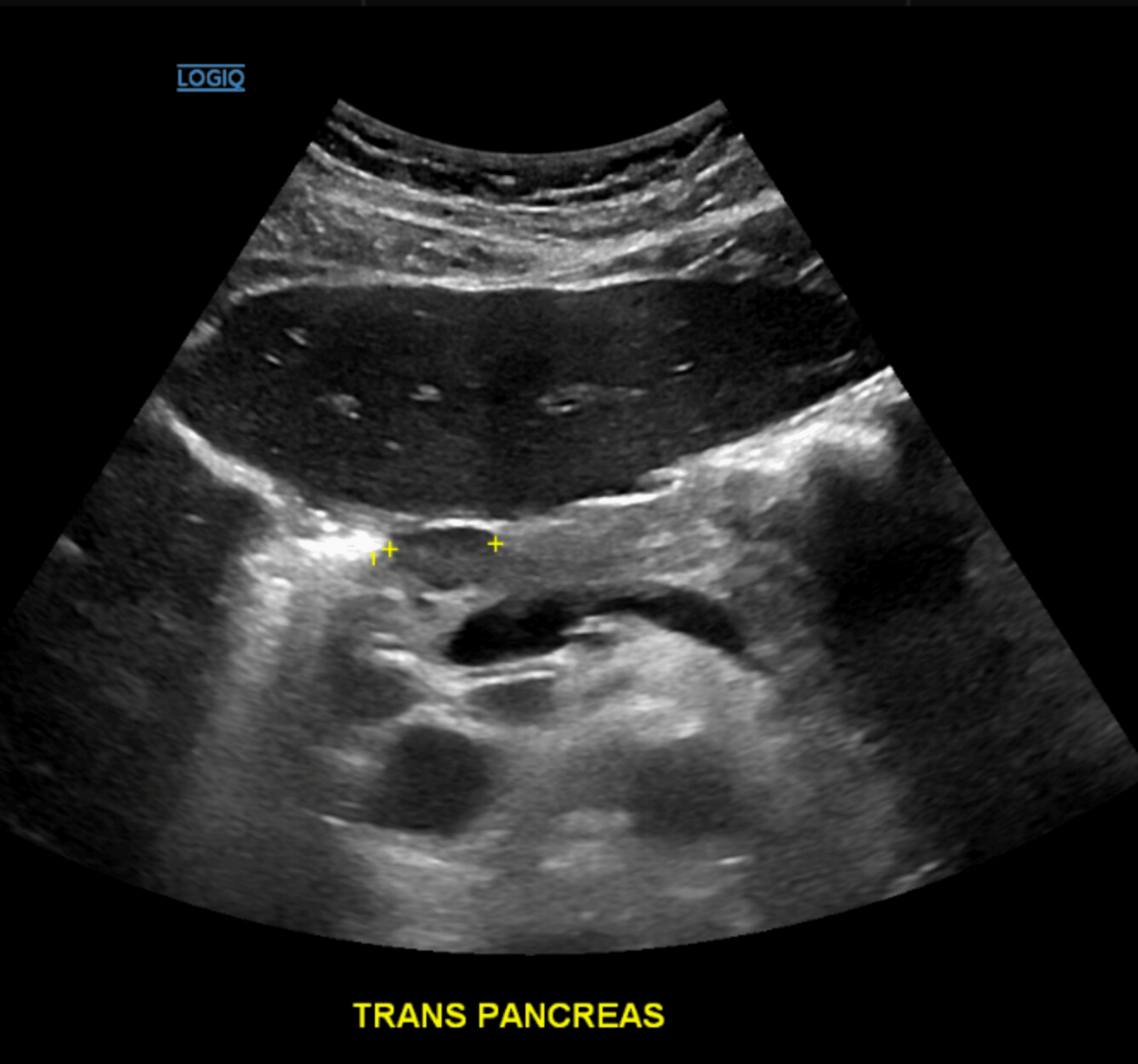
Abdominal ultrasound demonstrating a nodular density at the pancreatic head, shown by yellow markings on the image

**Figure 3:**
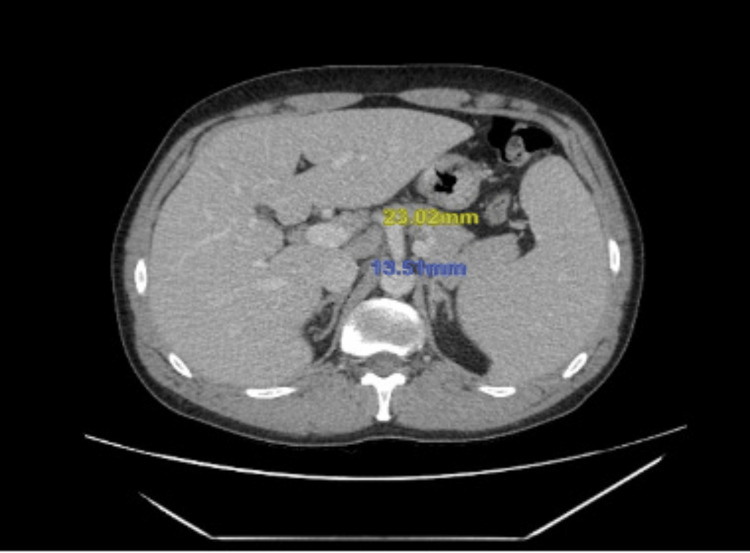
Computed tomography of the abdomen and pelvis demonstrating enlarged mesenteric lymph nodes, as marked by the yellow and blue numerical values

**Figure 4:**
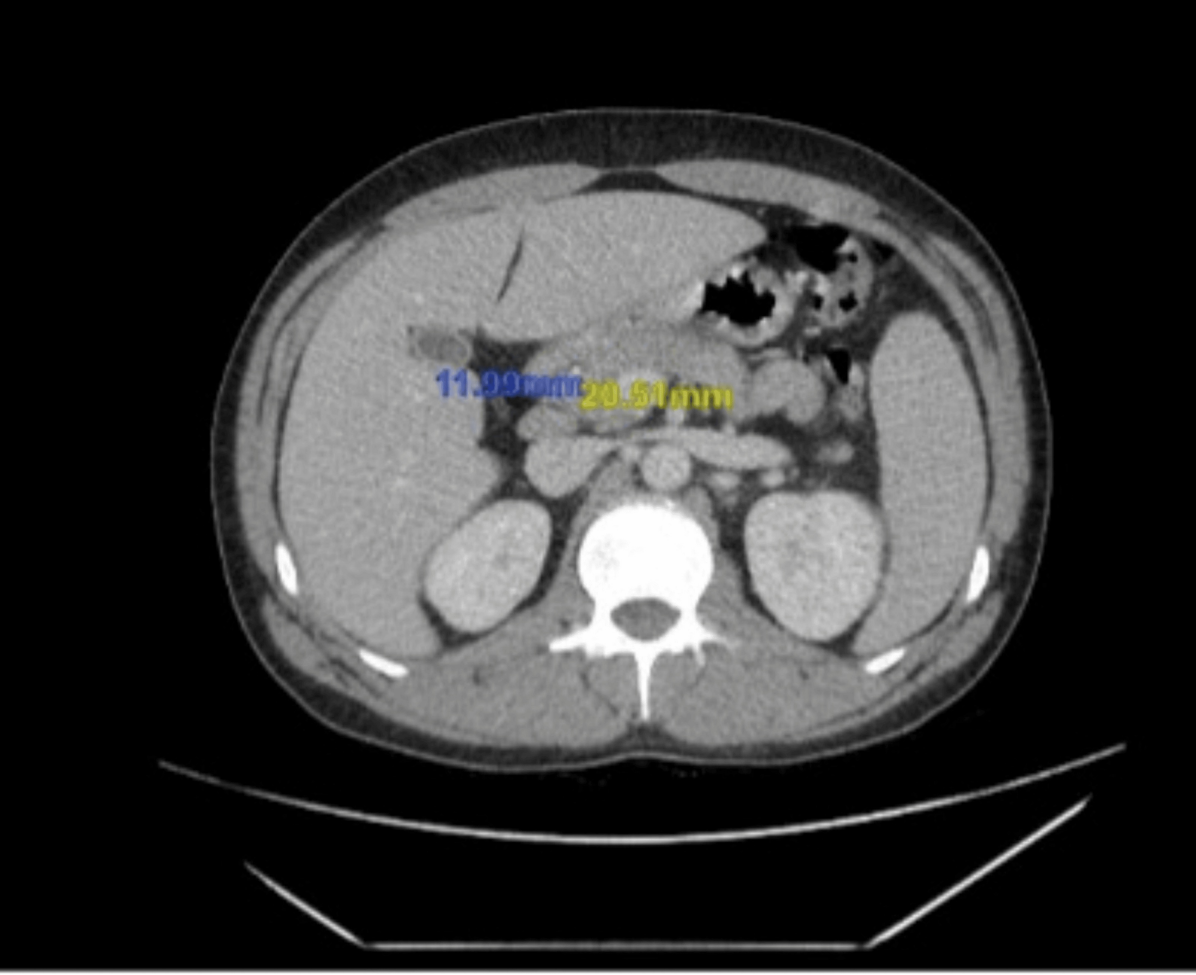
Computed tomography of the abdomen and pelvis demonstrating enlarged periportal and portocaval lymph nodes, as marked by the yellow and blue numerical values

Computed tomography of the abdomen and pelvis was remarkable for moderate splenomegaly (Figure 2) and diffuse lymphadenopathy involving lymph nodes of the porta hepatis, retroperitoneal, mesenteric, and bilateral iliac and inguinal regions (Figures 3, 4).

An extensive workup with EBV/CMV titers, tumor markers (e.g., CEA, CA 19-9, and AFP), and autoimmune titers was sent. While awaiting results, magnetic resonance cholangiopancreatography (MRCP) with and without contrast was obtained due to a nodular lesion seen on abdominal ultrasound, which showed a 14 cm enlarged spleen and multiple enlarged lymph nodes. There was no evidence of a pancreatic mass. CEA and AFP were negative. CA 19-9 was most likely falsely elevated at 48 due to systemic inflammation. Given extensive lymphadenopathy, a computed tomography of the chest with IV contrast was obtained, which showed a few prominent bilateral axillary lymph nodes and distal paraoesophageal lymph nodes.

**Figure 5:**
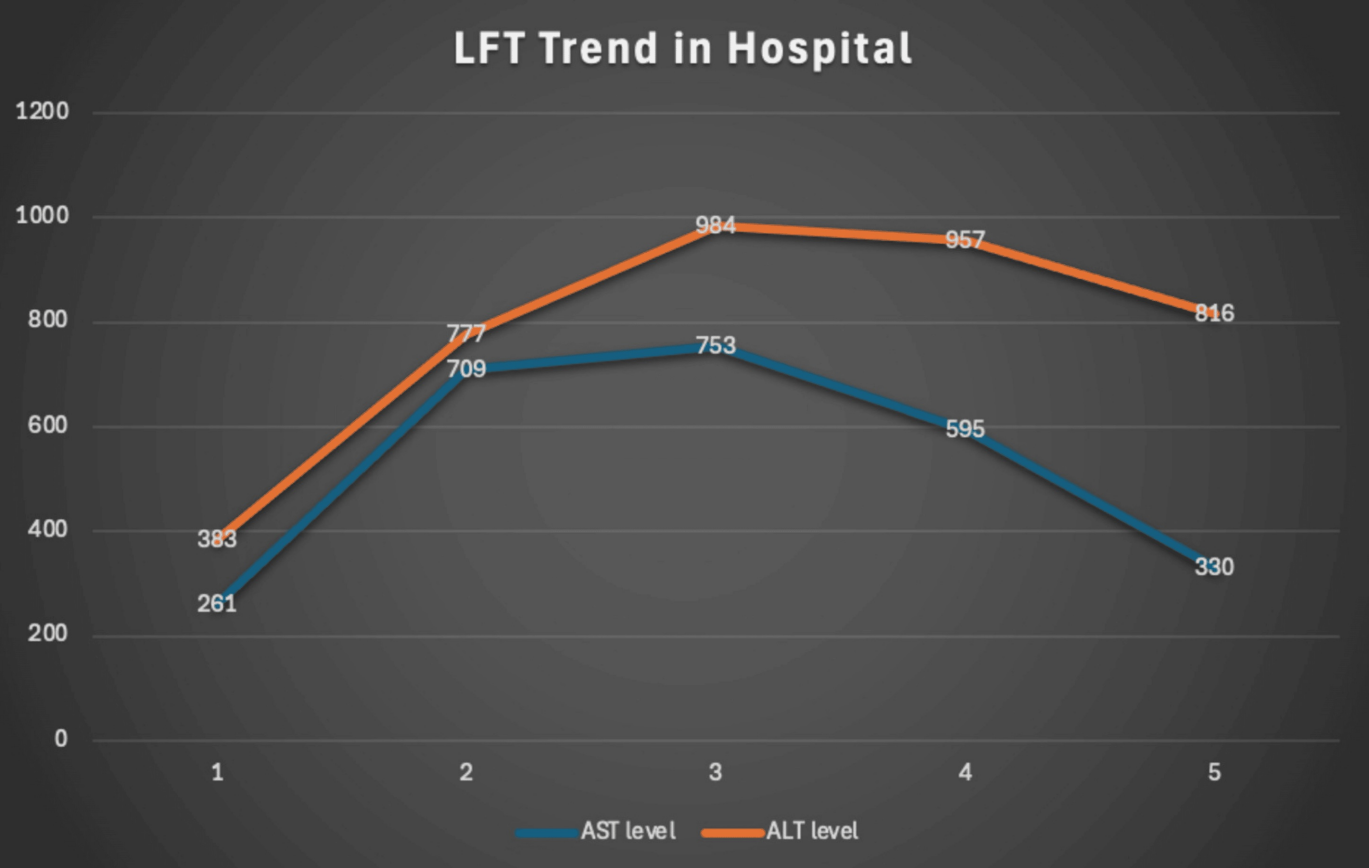
Liver function test (LFT) trend during hospital course with declining values post NAC treatment AST: Aspartate transferase; ALT: Alanine transaminase; NAC: N-acetylcysteine.

On day two of hospitalization, the patient’s liver function tests (LFTs) were more than doubled from the initial presentation (Table 2, Figure 5). Platelet count, PT, and INR remained stable and within normal levels. The patient noted the return of his dysphagia. Physical examination revealed continued scleral icterus and jaundice below the tongue; however, the patient was noted to have right cervical adenopathy with tenderness and multiple thin papilliform-like lesions on the tongue.

**Table 2:** EBV IgG and IgM values demonstrating acute EBV infection EBV: Epstein-Barr virus.

Laboratory Marker	Laboratory Value	Normal Range
EBV VCA IgG	25.7 U/mL	0–17.9 U/mL
EBV VCA IgM	96.2 U/mL	0–35.9 U/mL

A monospot test that was sent resulted as positive. EBV titers also resulted, demonstrating acute EBV infection (Table 3). CMV titers were within normal limits. Repeat LFTs later that day revealed further significant elevation (Table 2, Figure 5). The case was discussed with the intensivist team, and given the significant rise in the LFTs and concern for progression of hepatitis, it was decided to begin treatment with N-acetylcysteine. The patient received a loading dose of 16,000 mg, followed by another dose at 8,000 mg.

**Table 3:** The trend of liver function tests over the course of hospitalization AST: Aspartate transferase; ALT: Alanine transaminase.

Hospitalization Day	AST Level	ALT Level
Day 1	261 IU/L	383 IU/L
Day 2 AM	709 IU/L	777 IU/L
Day 2 PM (initiation of NAC treatment)	753 IU/L	984 IU/L
Day 3	595 IU/L	957 IU/L
Day 4	330 IU/L	816 IU/L

On day three of hospitalization, his LFTs started to decline (Table 2, Figure 5). Bilirubin levels remained stable, and WBC was also downtrending. His scleral icterus and jaundice below the tongue showed improvement. The papilliform lesions on the tongue increased in number. Cervical adenopathy became bilateral.

Over the course of the hospitalization, the LFTs remained downtrending (Table 2, Figure 5). Total bilirubin was also downtrending. WBC remained stable. The patient’s left upper quadrant pain resolved. Scleral icterus was minimal. On presentation to the clinic the following week, the patient had complete resolution of symptoms and jaundice. Physical examination revealed no cervical adenopathy. Oral examination showed improvement of the papilliform lesions. LFTs and bilirubin were found to normalize on repeat testing.

## Discussion

EBV is the most common pathogen causing IM, and around 95% of healthy adults have been infected with it. While patients present mainly with flu-like symptoms, sore throat, and lymphadenopathy, the older the patient at primary infection, the more severe the presentation [6]. Besides deep kissing being the main route of transmission, blood transfusion and solid organ transplant also account for a few cases [3]. The classic symptoms are seen four to seven weeks after the incubation period, and in most cases, the disease is self-limiting with a good prognosis. In rare cases, however​​​​​​, complications such as airway obstructions due to tonsil enlargement, hepatitis, splenic rupture, EBV-associated hemophagocytic syndrome, hemolytic anemia, meningitis, neurological abnormalities, and interstitial nephritis can be seen [3,7]. Additionally, individuals who had EBV are more likely to have multiple sclerosis and Hodgkin’s lymphoma [3]. The mainstay of treatment is supportive care, along with fluids and analgesics. Treatments such as acyclovir and corticosteroids have not been proven to be beneficial [8].

While subclinical hepatitis can be seen in 75% of patients with IM​​​​​​, about 5%-10% of patients can present with jaundice due to liver parenchymal injury. Transaminitis has been shown to resolve within a few weeks, but cases of liver failure, even in immunocompetent patients, have been reported [7]. Although reports on the use of N-acetylcysteine (NAC) are scarce, NAC may prevent worsening of transaminitis [9]. In a 2015 and 2021 meta-analysis and a 2012 randomized control trial, NAC was found to improve overall survival, post-transplant survival, and transplant-free survival in patients with acute liver failure, regardless of the cause [10-12]. The latent membrane protein 1 (LMP1) of EBV causes inflammation that both progresses to premalignant and malignant cells and triggers cytokines that heighten the inflammatory response [5].

Since the 1970s, NAC has been used to treat liver failure due to acetaminophen. It neutralizes the free oxygen radicals and restores glutathione in the mitochondria and cytoplasm, maintaining cell integrity. It also has a vasodilatory and ionotropic role, allowing for perfusion to organs in a shock state [10]. NAC was also found, in vivo, to reduce the leukocyte load and inhibit the leukocyte recruitment in inflamed tissue [5]. The medication is well tolerated, with minimal adverse effects including arrhythmias, dizziness, and allergic reactions [10].

In the case we present, the patient developed severe viral hepatitis due to EBV. Although NAC is not a known treatment for viral hepatitis, recent meta-analyses and randomized trials have highlighted its potential, emphasizing its role as a therapeutic option with a favorable safety profile. Given this and the concern of progressive viral hepatitis in the patient due to recurrent symptoms and worsening LFTs, treatment was initiated. There were no imminent signs of liver failure given the normal INR and platelet levels. The patient’s transaminitis normalized one week after treatment. The challenge, however, is determining if the resolution of the viral hepatitis was actually due to the use of NAC or just the natural course of the EBV hepatitis. The argument could be made that it was rather the former, given the decline in the patient's LFTs and symptomatic improvement during and shortly after treatment.

Overall, this abstract underscores the evolving landscape of NAC as a potential treatment modality in viral hepatitis-induced liver injury, shedding light on its promise in improving outcomes and mitigating liver damage in patients with progressive viral hepatitis and even potentially acute liver failure. With minimal adverse effects, the use of NAC in viral hepatitis merits further exploration.

## Conclusions

EBV hepatitis is a rare cause of viral hepatitis. We acknowledge that our patient did not meet established criteria for acute liver failure, and that most cases of EBV-associated hepatitis are self-limiting with supportive care alone. Therefore, a direct causal relationship between NAC administration and clinical improvement cannot be definitively established in this single case. Rather than implying a clear treatment effect, this report should be interpreted as hypothesis-generating. NAC may represent a reasonable adjunctive therapy in select patients with severe or progressive EBV hepatitis given its favorable safety profile; however, further studies are needed before routine use can be recommended.
